# Activation of Orexin 1 Receptors in the Paraventricular Nucleus Contributes to the Development of Deoxycorticosterone Acetate-Salt Hypertension Through Regulation of Vasopressin

**DOI:** 10.3389/fphys.2021.641331

**Published:** 2021-02-03

**Authors:** Jeremy A. Bigalke, Huanjia Gao, Qing-Hui Chen, Zhiying Shan

**Affiliations:** ^1^Department of Kinesiology and Integrative Physiology, Michigan Technological University, Houghton, MI, United States; ^2^Department of Psychology, Montana State University, Bozeman, MT, United States; ^3^The Second Clinical College of Guangzhou University of Chinese Medicine, Guangzhou, China; ^4^Health Research Institute, Michigan Technological University, Houghton, MI, United States

**Keywords:** orexin, hypertension, deoxycorticosterone acetate, vasopressin, paraventricular nucleus, blood pressure

## Abstract

Salt-sensitivity is a major factor in the development of hypertension. The brain orexin system has been observed to play a role in numerous hypertensive animal models. However, orexin’s role in the pathology of salt-sensitive hypertension (SSH) remains to be adequately explored. We assessed the impact of orexin hyperactivity in the pathogenesis of the deoxycorticosterone acetate (DOCA) – salt rat model, specifically through modulation of Arginine Vasopressin (AVP). Adult male rats were separated into three groups: vehicle control, DOCA-salt, and DOCA-salt+OX1R-shRNA. DOCA-salt rats received subcutaneous implantation of a 21-day release, 75 mg DOCA pellet in addition to saline drinking water (1% NaCl and 0.2% KCl). DOCA-salt+OX1R-shRNA rats received bilateral microinjection of AAV2-OX1R-shRNA into the paraventricular nucleus (PVN) to knockdown function of the Orexin 1-Receptor (OX1R) within that area. Following 2-week to allow full transgene expression, a DOCA pellet was administered in addition to saline drinking solution. Vehicle controls received sham DOCA implantation but were given normal water. During the 3-week DOCA-salt or sham treatment period, mean arterial pressure (MAP) and heart rate (HR) were monitored utilizing tail-cuff plethysmography. Following the 3-week period, rat brains were collected for either PCR mRNA analysis, as well as immunostaining. Plasma samples were collected and subjected to ELISA analysis. In line with our hypothesis, OX1R expression was elevated in the PVN of DOCA-salt treated rats when compared to controls. Furthermore, following chronic knockdown of OX1R, the hypertension development normally induced by DOCA-salt treatment was significantly diminished in the DOCA-salt+OX1R-shRNA group. A concurrent reduction in PVN OX1R and AVP mRNA was observed in concert with the reduced blood pressure following AAV2-OX1R-shRNA treatment. Similarly, plasma AVP concentrations appeared to be reduced in the DOCA-salt+OX1R-shRNA group when compared to DOCA-salt rats. These results indicate that orexin signaling, specifically through the OX1R in the PVN are critical for the onset and maintenance of hypertension in the DOCA-salt model. This relationship is mediated, at least in part, through orexin activation of AVP producing neurons, and the subsequent release of AVP into the periphery. Our results outline a promising mechanism underlying the development of SSH through interactions with the brain orexin system.

## Introduction

Hypertension is a major pathological condition that impacts roughly one-third of adults in the United States, with implications in the development of further cardiovascular complications such as cardiac ischemia, heart failure, and stroke (Centers for Disease and Prevention, 2011). Together, these conditions comprise the leading cause of death among adults in the United States. Further studies have shown that the majority of hypertensive individuals are observed to have impaired sodium handling, resulting in enhanced pressor response following a salt challenge (Weinberger et al., 1986; Weinberger, 1996; Choi et al., 2015). This condition is known as salt-sensitive hypertension (SSH) and is estimated to effect approximately half of individuals with diagnosed primary hypertension, not including those individuals who remain undiagnosed (Weinberger, 1996; Choi et al., 2015). As salt intake increases, the relevance of this disease has become more apparent, leading numerous researchers to assess the underlying mechanisms mediating this hypertensive response.

Since its discovery in 1998 by two groups concurrently (de Lecea et al., 1998; Sakurai et al., 1998), orexin has come under the spotlight as a potential regulator of feeding, sleep/wake cycles, in addition to blood pressure regulation. Orexin A (OXA) and orexin B (OXB) are the two subtypes of orexin neuropeptides derived from a single precursor, and while they both carry out their functions on G protein coupled receptors Orexin Receptor 1 (OX1R) and Orexin Receptor 2 (OX2R), OX1R has a much higher affinity for OXA than OXB. Although OXA and OXB producing neurons are found solely in the lateral hypothalamic area (LHA; de Lecea et al., 1998; Sakurai et al., 1998), their neuronal projections densely innervate numerous areas of the brain including the hypothalamus, brain stem, limbic system, as well as the circumventricular organs (CVOs; Peyron et al., 1998; Nambu et al., 1999; Kilduff and Peyron, 2000). Interestingly, orexin producing neurons densely innervate their respective receptors in the paraventricular nucleus (PVN; Trivedi et al., 1998; Li and Nattie, 2014), a major area of cardiovascular control. Samson et al. (1999) were some of the first to observe a dose-dependent increase in mean arterial pressure (MAP) following intracerebroventricular (ICV) injection of OXA and OXB in Sprague Dawley (SD) rats. Others confirmed these results, but also observed an increase in sympathetic nerve activity (SNA) following injection, indicating that orexin exerts its influence on the cardio vasculature through sympathetic activation (Shirasaka et al., 1999; Li and Nattie, 2014). Further, Schwimmer et al. (2010) observed an attenuated resting blood pressure following genetic knockdown mRNA of the orexin precursor, prepro-orexin. The observed role of orexin hyperactivity in hypertension were replicated in subsequent studies in animal models of primary hypertension including the BPH/2J mouse (Marques et al., 2011), and the Spontaneously Hypertensive rat (SHR; Lee et al., 2013, 2015; Li et al., 2013), where central or orally administered orexin receptor antagonists resulted in attenuated blood pressure responses.

To the best of our knowledge, so far, no study has been conducted assessing the role of orexin in the development of SSH in other group. Our recent studies have shown that proinflammatory cytokine (PIC) activity is augmented in the PVN of Dahl-Salt (Dahl-S) Sensitive rats, but not in normal SD rats (Fan et al., 2018). This effect was similarly observed following PVN injection of OXA in SD rats, which resulted in heightened sympathetic outflow. Furthermore, Huber et al. (2017) observed that a high-salt diet in Dahl-S rats results in increased central orexin signaling, in addition to Arginine Vasopressin (AVP) production, resulting in an increase in blood pressure. Importantly, this effect was attenuated following OX1R blockade in the PVN, while sympathetic outflow remained unchanged (Huber et al., 2017). The significant reduction in blood pressure, but not sympathetic outflow, indicates that orexin may regulate SSH development through regulation of AVP.

The importance of the PVN in AVP production and cardiovascular regulation has led us to believe that this may be the primary pathway utilized in orexin regulation of SSH. However, in order to extend and replicate this work, further SSH rat models must be utilized. Deoxycorticosterone acetate (DOCA) administration to SD rats, in addition to salt intake has been shown to elicit an increase in blood pressure, which is maintained at a heightened level, specifically through AVP overactivity (Yu et al., 2001). This model is also marked by enhanced Renin-Angiotensin-System (RAS) activity, resulting in endocrine dysfunction, as well as sympathetic hyperactivity. Only one study to date has assessed orexin system activity in DOCA-salt rats. Hernandez et al. (2018) observed increased expression of orexin components within the hypothalamus, similar to our own results. However, this study did not assess the impact of orexin receptor activity in the PVN, nor did it review AVP as a potential mediator of the hypertensive response observed in the DOCA-salt model.

To this end, the present study aims to assess the role of PVN orexin overactivity in the pathology of DOCA-salt hypertension with the hypothesis that overactive orexin activity within the PVN will result in greater PVN AVP expression as well as blood AVP release, and increased blood pressure. Further, we hypothesize that knockdown of OX1R function in the PVN will attenuate this pressor response.

## Materials and Methods

### Animals

All rats used in this study were purchased from Charles River Laboratories (Wilmington, MA). Adult male rats (200–350 g) were placed into three groups: DOCA-salt, DOCA-salt+OX1R-shRNA, and vehicle control. The DOCA-Salt group received implantation of a 21-day release DOCA pellet (75 mg, Innovative Research of America, FL, United States), and saline drinking solution (1% NaCl and 0.2% KCl) with normal chow for 21 days. DOCA-salt+OX1R-shRNA rats received a PVN microinjection of AAV2-OX1R-shRNA (University of Florida) 2 weeks prior to DOCA and high salt diet administration, identical to the DOCA-Salt group. We elected to omit uninephrectomy from the procedure in order to attain a more gradual, modest increase in blood pressure that more closely mimics the development observed in humans (Kandlikar and Fink, 2011). The vehicle controls were given sham surgeries for pellet implantation, and normal water and chow. During the 3 weeks of treatment, blood pressure measurements were taken on DOCA-salt, DOCA-salt+OX1R-shRNA, and control rats. Following this, rats were euthanized and used for PCR, immunostaining, plasma, and heart weight measurements. The time of euthanization was confined to early afternoon, between approximately 2:00 and 5:00 pm, in order to mitigate the chance of any variability in measured physiological parameters due to the natural fluctuations of orexin activity throughout the day. All rats were housed at a constant temperature and a 12:12 h light dark cycle and given their respective diets ad libitum. All animal experiments were performed in adherence to protocols approved by the Michigan Technological University Institutional Animal Care and Use Committee (IACUC).

### Hypothalamic Paraventricular Nucleus Microinjections

Prior to any diet or DOCA treatment, DOCA-salt+OX1R-shRNA rats were subjected to bilateral PVN microinjection of AAV2-OX1R-shRNA. The AAV viral vector was packaged in the University of Florida Viral Vector Center. Briefly, a synthetic complementary DNA (cDNA) oligonucleotide encoding shRNA targeting OX1R mRNA (CTACTTCATTGTCAACCTGT) was cloned into an AAV vector, PTR-UF11, under the control of human U6 promoter. A green fluorescence protein (GFP) reporter gene under the control of CBA promoter was cloned upstream of the shRNA expression cassette in order to directly visualize expression from the vector after delivery, generating construct AAV-OX1R-shRNA. The constructs were packaged into the AAV2, and virus production and titers were determined as previously described (Hauswirth et al., 2000). Rats were anesthetized using 5% isoflurane for induction, and 2–3% isoflurane exposure for maintenance of anesthesia. Following complete anesthesia, rat heads were fixed in a stereotaxic frame. Two holes were drilled through the skull at coordinates of the PVN so that a single glass microinjector pipette could be lowered into the PVN area. The coordinates for the PVN (in mm) were as follows: −1.6 caudal to bregma, 0.5–0.7 lateral to the midline, and 7.2 deep. Once the microinjector was in place, 200 nl of AAV2-OX1R-shRNA was injected bilaterally into the PVN. Approximately 10–12 min was taken between injections to allow for diffusion of the viral vector. Following injection, the wound was sutured, and rats were given a subcutaneous injection of a cocktail solution of meloxicam, penicillin, and sterile 0.9% saline the day of injection, as well as 2 days after in accordance with IACUC and ACF standards. DOCA-Salt OX1R-shRNA rats were given 2 weeks to recover as well as to allow full viral expression, before any other procedures were performed.

### DOCA Pellet Implantation

DOCA-salt and DOCA-salt+OX1R-shRNA groups were subjected to subcutaneous implantation of a DOCA pellet (75 mg, 21-day release, Innovative Research of America, FL, United States) prior to beginning the high salt drink treatment. Rats were given 5% isoflurane for anesthesia induction followed by 2–3% isoflurane to maintain adequate anesthesia during the procedure. An incision was made in the retro-scapular region, and the DOCA pellet was placed subcutaneously. The wound was sutured, and rats were given the same post-operative care as above. However, directly following the procedure, all rats’ drinking water was switched to a saline solution (1% NaCl and 0.2% KCl) for the remainder of the 21 days. As previously mentioned, DOCA treatment is usually paired with uninephrectomy to exacerbate the development of hypertension. However, we decided to exclude the kidney removal following a previously established model (Kandlikar and Fink, 2011) to obtain a more gradual progression of hypertension development that more closely mimics that seen in a humans, as well as to eliminate many of the adverse effects that traditional DOCA-salt and uninephrectomy incurs (Gavras et al., 1975; Wada et al., 1995; Loch et al., 2006).

### Blood Pressure Measurement

A subset of each group was subjected to blood pressure measurement during their treatment period using tail plethysmography (Kent Scientific, CT, United States). DOCA-salt, DOCA-salt+OX1R-shRNA, and control rats were all acclimated to the procedure for a week before measurements began through everyday blood pressure measurements, as recommended by the American Heart Association (Kurtz et al., 2005). Briefly, rats were placed in a plastic cylinder with a dark nose-cone, to reduce vision and anxiety. After 10 min of acclimation, the tail cuff apparatus including the occlusion cuff and volume pressure recording cuff were placed on the animal for an additional 5–10 min. Artificial heating was also applied to maintain adequate blood flow to the tail. Following this, blood pressure recording began, and 10 acclimation cycles followed by 20 measurement cycles were conducted. Following blood pressure recording, averages of the 20 measurement cycles were taken and paired with their group to obtain a group mean, which was utilized for statistical analysis. Rats maintained acclimation during the 18-day treatment by daily 20-min sessions in the rat holder/tail cuff apparatus for every rat, to reduce variability and anxiety among the rats.

Tail-cuff plethysmography can be attributed to stress-related fluctuations in blood pressure, due to confinement in a small cylinder for extended periods of time, which may lead to variability of results. However, all necessary precautions were taken to mitigate this effect. Namely, acclimation to the cylinders as well as the occlusion/volume pressure recording apparatus began 2 weeks prior to any actual blood pressure recording was performed. Following 1 week of acclimation, blood pressure measurements were taken to identify when the blood pressure reached a normotensive level in all treatment groups. Once the blood pressure appeared to maintain a consistent, normotensive pressure, the experiment was initiated. In addition, blood pressure recording sessions were done during the same time every day (12:00–4:00 pm), to mitigate circadian influences on orexin system function, and thus to alleviate any fluctuations in blood pressure that may naturally occur with differential orexin system activity throughout the day. Furthermore, during the 3 weeks of blood pressure recording, acclimation was maintained on days when blood pressure measurements were not taken by daily exposure to both confinements in the cylinder as well as attachment of the occlusion/volume pressure recording apparatus for 20 min.

### Real-Time PCR and Immunostaining

Following 3 weeks of treatment, rats were euthanized and subjected to either real time polymerase chain reaction (PCR) analysis to assess mRNA expression or immunostaining to visualize protein levels of the genes of interest in the PVN. For PCR, brains were removed and immediately flash frozen in liquid nitrogen. Following flash freezing, all brains were placed into a −80°C freezer, where they would remain until needed for mRNA analysis. Upon removal from the freezer, the PVN area was punched. RNA was isolated using RNeasy plus Mini kits (Qiagen, CA, United States) following manufacturer’ instructions. Following isolation, ~200 ng RNA from each sample was converted to cDNA in 20 μl PCR system using iScript cDNA synthesis kits (Bio-Rad), and the cDNAs were used as templates for real time PCR, which was performed to analyze mRNA levels of prepro-orexin (a precursor of OXA and OXB), OX1R, and AVP using gene specific TaqMan primers and probes. TaqMan primers were purchased from Fisher Scientific and were used in real-time PCR to measure mRNA levels of prepro-orexin (Hcrt), OX1R, AVP, and a housekeeping gene, GAPDH, which was used as an endogenous control. The manufacturer did not provide the detailed primer sequence information. The primer IDs were as follows: Hcrt: Rn00565995_m1; OX1R: Rn00565032_m1; AVP: Rn00566449_m1; and GAPDH: Rn01775763_g1.

Immunostaining was performed to analyze OX1R, OXA, and AVP protein expression within the PVN as described previously (Huber et al., 2017). Briefly, rats were deeply anesthetized using isoflurane. Once under deep anesthesia, cold phosphate buffer saline (PBS) followed by 4% paraformaldehyde (PFA) in 1xPBS was used to transcardially perfuse the animal. Following perfusion, the brain was removed and kept in 4% PFA overnight in 4°C. The next day, the brains were transferred and kept in 30% sucrose at 4°C until they sank to the bottom. Brains were then cut in 20-μm coronal sections using a cryostat. Brain sections containing the PVN area were then subjected to immunostaining. Following wash in 1xPBS 3 times for 10 min each, brain sections were incubated with either rabbit anti-OX1R antibody (Alomon Laboratories, Jerusalem, Israel, 1:300 dilution), rabbit anti-AVP antibody (OriGene Technologies, United States, 1:400 dilution), or mouse anti-OXA antibody (Abcam, United States, 1:300 dilution) in PBS containing 0.5% Triton X-100 and 5% horse serum for 72 h at 4°C. Following this incubation and 1xPBS washing, they were incubated overnight in secondary antibodies Alexa fluor 488 goat anti rabbit IgG (1:500), Alexa fluor 594 goat anti rabbit IgG (1:500), or Alexa fluor 594 donkey anti mouse IgG (1:500). Images representing immunofluorescence were taken with a Leica DMIL microscope.

### Plasma AVP ELISA Testing

Rats were placed under heavy anesthesia using 5% isoflurane. They were then decapitated, and their blood was collected into the tubes coated with K2EDTA. The final EDTA concentration is ~1 mg/ml blood. The blood was then centrifuged at 4°C using an Eppendorf 5,804 R Centrifuge at 1,300 RPM for 30 min. The supernatant was extracted and stored in −80°C until use. Plasma AVP level were measured using Arg8-Vasopressin Kit (Enzo Life Sciences, NY, United States) following the manufacturer’s instructions.

### Data Collection

All rats subjected to PFA perfusion and subsequent immunostaining were unable to be used for any physiological analysis other than protein immunofluorescence staining and blood pressure monitoring. However, all other animals subjected to brain and blood collection were utilized for multiple tests, including PCR and ELISA. Because of the overlap, and the multiple utilizations of one sample for various tests, some discrepancies in sample size may be present. In general, an arbitrary sample size of 5~9, based on our previous work regarding the role of orexin and AVP in SSH (Huber et al., 2017), was chosen as a goal for each treatment group. In addition, all animals that were lost during surgery or before any measurements were taken, were not recorded, and were simply replaced, eliminating any effect on sample size.

### Statistical Analysis

All data was analyzed using commercially available statistical software (SPSS 25.0, SPSS, Chicago). All data is expressed as mean ± SEM unless otherwise noted. Due to the directional nature of our hypothesis, and previous work from our lab, a one-tailed *t*-test was utilized to compare mean OX1R mRNA and AVP mRNA expression, as well as plasma AVP levels between control and DOCA-salt groups. Differences in mRNA expression and plasma concentrations between all three groups were analyzed using one-way ANOVA. If a significant interaction was observed, Tukey *post-hoc* analysis was utilized to assess group differences. Individual blood pressure means were measured for each rat three times per week at week 1, and week 2 measurements, while only one measurement was taken during week 3. For each rat, the three weekly measurements were averaged, and then combined with the other rats in the respective group to obtain a weekly group average blood pressure. These weekly averaged blood pressures were compared to the baseline values for each group (recorded values 1 day prior to treatment). For blood pressure and heart rate (HR) data, a repeated measures ANOVA was performed with time as the within factor, and treatment condition as the between. If significance was observed, a Tukey *post-hoc* analysis was performed between groups. All values with a *p* < 0.05 were considered significant.

## Results

### DOCA-Salt Treatment Increases PVN OX1R Expression and Elevates Plasma AVP Levels

**Figure 1:**
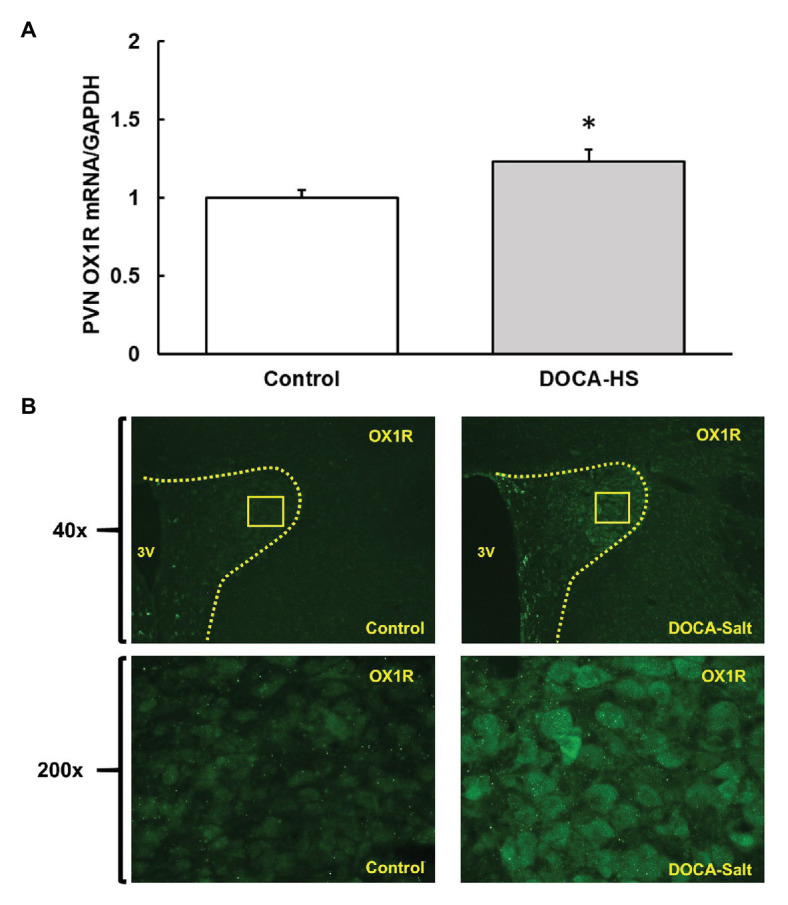
Deoxycorticosterone acetate (DOCA)-salt treatment increases orexin 1-receptor (OX1R) expression in the paraventricular nucleus (PVN) of Sprague Dawley (SD) rats. OX1R mRNA **(A)** and immunofluorescence **(B)** was compared in the PVN area between DOCA-salt treated (*n* = 4) and vehicle control (*n* = 3) rats. DOCA-salt treated rats showed a significant increase in OX1R expression within the PVN area (^*^p < 0.05). PVN, paraventricular nucleus; OX1R, orexin 1-receptor.

To assess whether OX1R expression was elevated in the PVN of DOCA-Salt rats, adult male SD rats were randomly divided into two groups. They were either subcutaneously implanted with a 75 mg, 21-day released DOCA pellet and received saline drinking water (1% NaCl and 0.2% KCl) or were subjected to sham surgeries and received regular drink water. Three weeks following DOCA-salt or sham treatment, rats were euthanized, and their brains were removed, and the PVN area was punched out and subjected to RNA isolation and subsequent real time PCR to measure OX1R mRNA expression. The results showed that PVN OX1R mRNA levels were significantly increased in the DOCA-salt treatment group (*n* = 4) compared to control animals [*n* = 3; Figure 1A, Control: 1 ± 0 vs. DOCA-salt: 1.2 ± 0.1, arbitrary units (a.u) *p < 0.05].

Additionally, brain slices containing the PVN area were utilized to perform immunostaining using OX1R specific antibodies in order to visually assess any differences in receptor expression in the brains of control and DOCA-salt rats. It showed that DOCA-salt treatment increased OX1R immunoreactivity in the PVN (Figure 1B).

**Figure 2:**
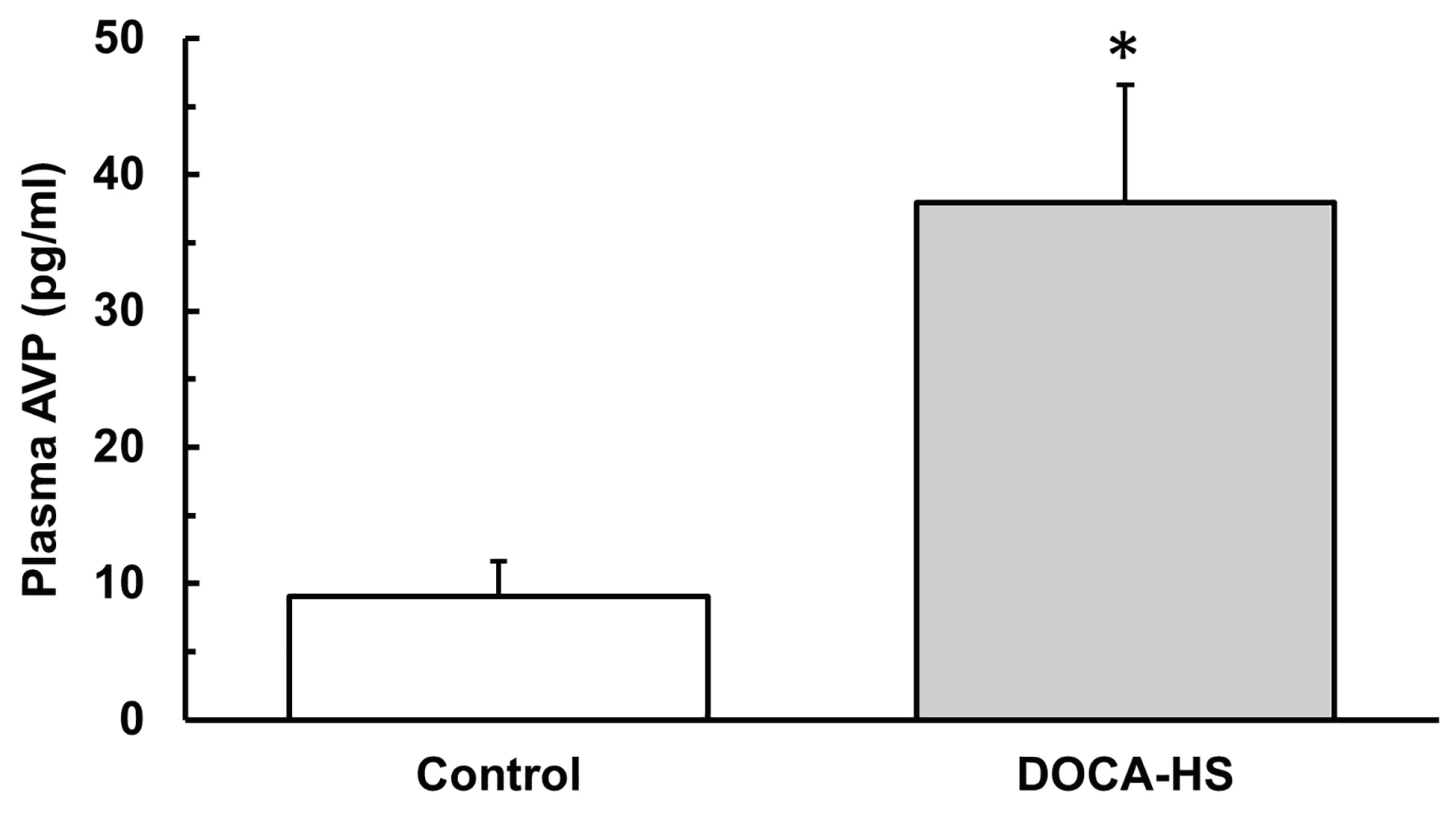
Deoxycorticosterone acetate-salt treatment significantly increases plasma arginine vasopressin (AVP) concentrations in SD rats. Following 3 weeks of DOCA-salt treatment or vehicle control, ELISA analysis was performed on the plasma of DOCA-salt (*n* = 8) and vehicle control (*n* = 4) rats to confirm the effectiveness of DOCA-salt treatment in causing increased plasma AVP. DOCA-salt rats showed a significantly increased plasma AVP concentration (^*^p < 0.05). AVP, arginine vasopressin.

We further performed ELISA testing to investigate whether DOCA-salt treatment elicited an increase in plasma AVP concentrations. The results showed that, following 3-weeks of DOCA-Salt treatment, AVP plasma levels were significantly elevated in the DOCA-Salt rats (*n* = 8) compared to control rats (*n* = 4; Figure 2, Control: 9 ± 3 vs. DOCA-salt: 38 ± 9 pg/ml, ^*^p < 0.05).

### Chronic Knockdown of OX1R in the PVN Attenuates Hypertension Development in DOCA-Salt Rats

**Figure 3:**
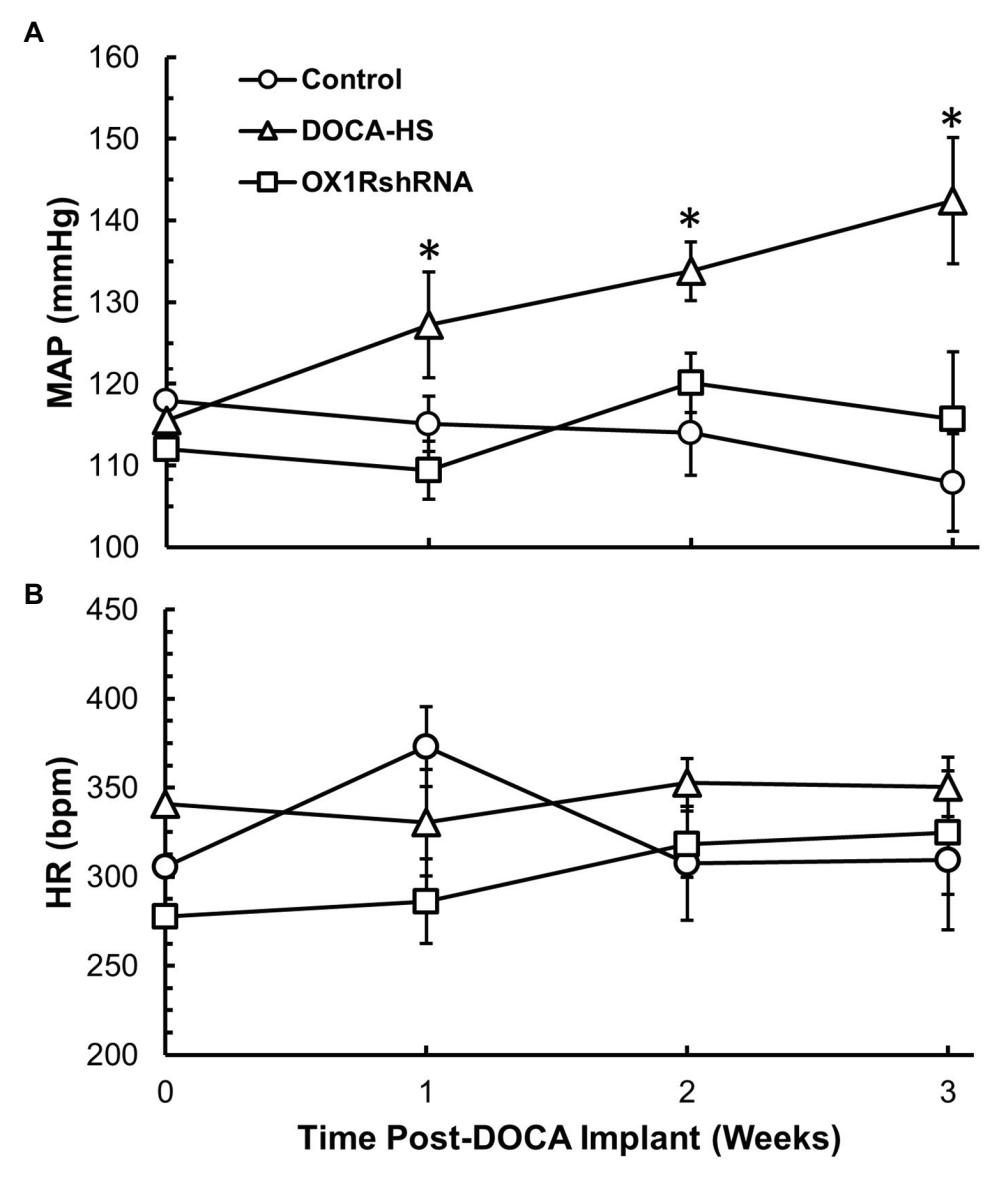
Chronic knockdown of OX1R in the PVN attenuates increased mean arterial pressure (MAP) induced by DOCA-salt treatment in SD rats. Rats were bilaterally injected with AAV2-OX1R-shRNA into the PVN 2 weeks prior to DOCA-salt treatment. Following this, blood pressure was monitored for 3 weeks during DOCA-salt treatment. Central knockdown of OX1R (*n* = 4) within the PVN results in attenuation of mean arterial pressure compared to DOCA-salt treated rats (*n* = 4; **A**; ^*^p < 0.05), while there was no difference between OX1R knockdown rats and controls (*n* = 5). OX1R knockdown within the PVN had no impact on heart rate between groups (**B**; *p* > 0.05). MAP, mean arterial pressure; HR, heart rate.

It is well established that DOCA-salt treatment can induce hypertension and increased AVP is implicated in this form of hypertension (Berecek et al., 1982a,b; Schenk and McNeill, 1992). Our aforementioned results indicate that DOCA-salt treatment results in a concurrent increase in both PVN OX1R expression as well as plasma AVP levels, indicating a potential causative role for orexin function in AVP secretion and hypertension development. In order to test the contribution of OX1R in the development of DOCA-salt hypertension, we tested whether chronic knockdown of PVN OX1R can prevent hypertension development. Male, 8-week-old SD rats were divided into three groups: DOCA-salt, DOCA-salt+OX1R-shRNA, and vehicle controls. Those in the DOCA-salt+OX1R-shRNA group received bilateral PVN microinjection of AAV2-OX1R-shRNA. Following sufficient time to allow transgene expression, animals began either DOCA pellet implantation followed by saline water treatment, or sham surgery followed by regular tap water drinking as detailed in the Methods. Their blood pressure was measured *via* tail-cuff plethysmography. There were no significant differences in baseline blood pressure (Control: 118 ± 4 vs. DOCA-Salt: 116 ± 2 vs. OX1R-shRNA: 112 ± 4 mmHg, *p* > 0.05) or HR (Control: 306 ± 28 vs. DOCA-Salt: 341 ± 58 vs. OX1R-shRNA: 278 ± 38 mmHg, *p* > 0.05) among groups. Our results showed that DOCA-salt treatment dramatically increased blood pressure, and chronic knockdown of PVN OX1R attenuated the observed increase in blood pressure induced by DOCA-salt treatment (Figure 3A). Specifically, a significant time × condition effect on MAP was observed [*F*(6,30) = 3.712, ^*^p < 0.05], indicating that there was differential MAP responsiveness over time among the three groups. There was also a significant group effect present [*F*(2,10) = 6.142, ^*^p < 0.05], indicating that the MAP response to treatment in each experimental group was significantly different over the 3-week period. Post-hoc analysis showed a significantly higher MAP in the DOCA-salt treatment group (*n* = 4) when compared to both the control (*n* = 5; ^*^p < 0.05) and OX1R-shRNA (*n* = 4; ^*^p < 0.05) groups. This effect was not observed in heart rate responses among groups [*F*(6,30) = 0.837, *p* > 0.05; Figure 3B].

**Figure 4:**
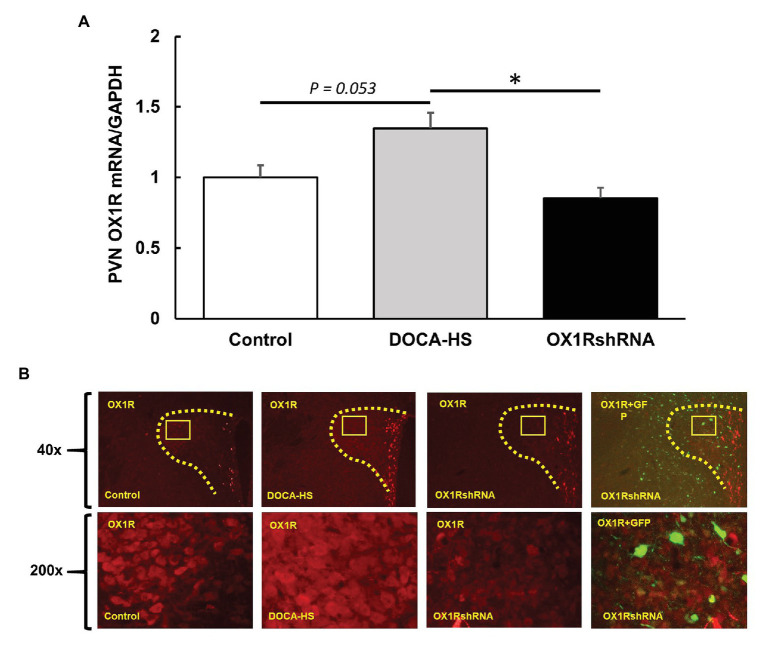
Paraventricular nucleus bilateral microinjection of AAV2-OX1R-shRNA significantly reduces OX1R expression within the brain. Following 3 weeks of DOCA-salt treatment, rat PVN areas were collected and subjected to either PCR analysis or immunostaining. mRNA levels of OX1R **(A)** were significantly reduced following OX1R knockdown (*n* = 5; ^*^p < 0.05) compared to DOCA-salt treated rats (*n* = 10). There were no significant differences between OX1R knockdown rats and controls (*n* = 7; *p* > 0.05). These results were further supported by immunostaining analysis **(B)**. PVN, paraventricular nucleus; OX1R, orexin 1-receptor.

We further compared OX1R mRNA expression in the PVN among the three groups of rats. There was a significant interaction between treatment condition and PVN OX1R mRNA levels [*F*(2,19) = 6.168, ^*^p < 0.05]. *Post-hoc* analysis showed that DOCA-salt (*n* = 10) treated rats showed an increase of approximately 35% in OX1R mRNA expression when compared to controls (*n* = 7; Control: 1 ± 0.1 vs. DOCA-salt: 1.3 ± 0.1, a.u, *p* = 0.053). This effect was attenuated in OX1R-shRNA animals (*n* = 5; OX1R-shRNA: 0.9 ± 0.1 vs. DOCA: 1.3 ± 0.1, a.u, ^*^p < 0.05). There were no significant differences between control and OX1R-shRNA animals (*p* = 0.653; Figure 4A).

Similarly, control, DOCA-salt, and DOCA-salt+OX1R-shRNA animals’ brains were subjected to OX1R immunostaining as described previously to visually assess the adequacy of AAV-OX1R-shRNA in knocking down OX1R function. The mRNA results were similarly mirrored in immunostaining results shown in Figure 4B. The combination of these results indicates that our viral vector sufficiently knocked down OX1R expression in the PVN and decreased PVN OX1R attenuated DOCA-salt induced high blood pressure.

### Chronic Knockdown of PVN OX1R Reduces PVN AVP Expression and Partially Reduces Plasma AVP Levels

**Figure 5:**
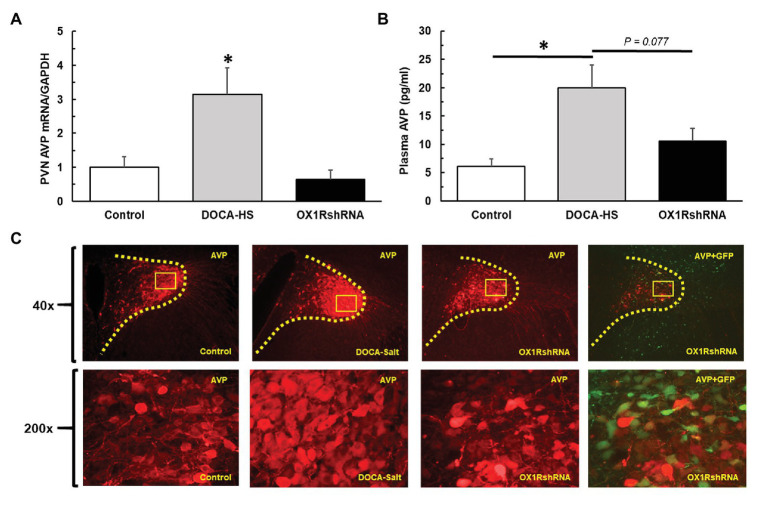
Paraventricular nucleus bilateral microinjection of AAV2-OX1R-shRNA significantly reduces central production and subsequent peripheral release of AVP. Following 3 weeks of DOCA-salt treatment, brain PVN areas as well as plasma were collected to test AVP central expression and peripheral secretion. PVN OX1R knockdown (*n* = 5) resulted in a significantly reduced PVN AVP mRNA expression **(A)** compared to DOCA-salt treated rats (*n* = 9; ^*^p < 0.05), but not control rats (*n* = 8; *p* > 0.05). Similarly, OX1R knockdown in the PVN (*n* = 5) appeared to partially reduce plasma AVP levels compared to DOCA-salt rats (*n* = 6; **B**), although this did not reach significance (*p* = 0.077). OX1R knockdown rats showed no differences in plasma AVP levels when compared to control rats (*n* = 6; *p* > 0.05). Lastly, immunostaining revealed a reduced protein expression of AVP in the PVN area following OX1R knockdown **(C)**. PVN, paraventricular nucleus; AVP, arginine vasopressin.

We further assessed the effect of chronic knockdown of PVN OX1R on AVP expression in the PVN. DOCA-salt treatment significantly increased PVN AVP expression and chronic knockdown of OX1R blocked the AVP increase induced by DOCA-salt treatment. Figure 5A shows the differences in PVN AVP expression between groups. There was a significant interaction between treatment condition and PVN AVP mRNA levels [*F*(2,19) = 5.085, ^*^p < 0.05]. *Post-hoc* analysis showed that DOCA-salt (*n* = 9) treated rats showed a significantly increased AVP mRNA when compared to controls (*n* = 8; Control: 1 ± 0.3 vs. DOCA-salt: 3.1 ± 0.8, a.u, ^*^p < 0.05). However, this effect was attenuated in OX1R-shRNA animals (*n* = 5; OX1R-shRNA: 0.6 ± 0.3 vs. DOCA: 3.1 ± 0.8, a.u, ^*^p < 0.05). There were no significant differences between control and OX1R-shRNA animals (*p* > 0.05).

We further measured and compared plasma AVP levels of the three groups of rats to assess whether the observed increase in PVN production of AVP translated to increased peripheral circulation of AVP. ANOVA analysis revealed a significant difference in plasma AVP levels between groups [*F*(2,13) = 7.02, ^*^p < 0.05]. *Post hoc* analysis revealed that DOCA-salt (*n* = 5) treated rats presented a significantly increased plasma AVP concentration when compared to controls (*n* = 6; Control: 6 ± 1 vs. DOCA-salt: 20 ± 4 pg/ml, ^*^p < 0.05). Additionally, OX1R-shRNA rats (*n* = 5) had a partially attenuated plasma AVP level (11 ± 2 pg/ml) compared to DOCA-salt rats, although this relationship only trended toward significance (*p* = 0.077; Figure 5B).

Consistent with this finding in AVP mRNA expression and plasma AVP, immunostaining showed that DOCA-salt treatment drastically increased AVP immunoreactivity in the PVN, and chronic knockdown of OX1R in the PVN blocked DOCA-salt induced increases in PVN AVP protein expression Figure 5C.

## Discussion

**Figure 6:**
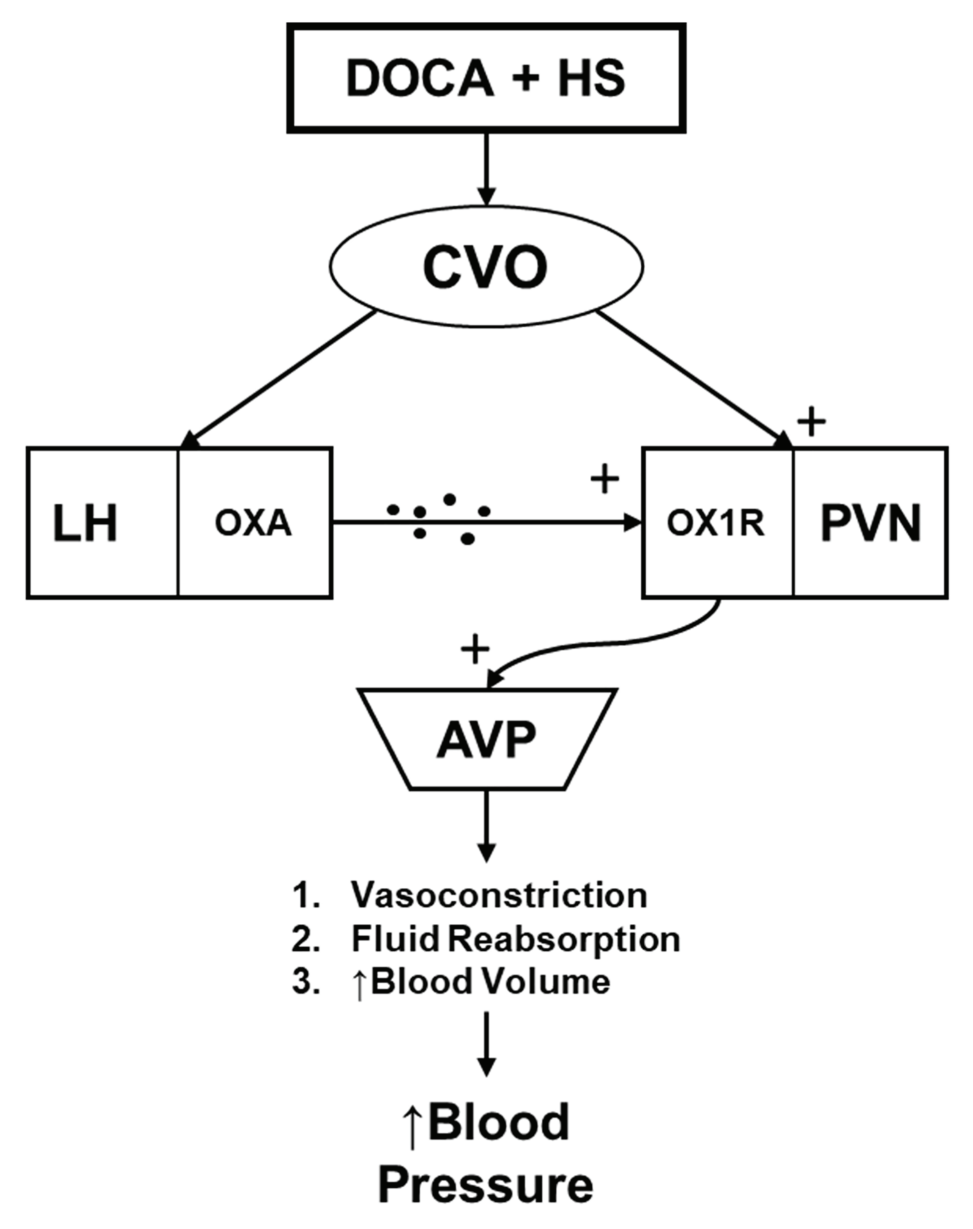
The hypothesized relationship mediating the impact of central orexin system functioning on hypertension development in the DOCA-salt rat model. DOCA-salt treatment results in activation of circumventricular organs (CVOs), which send projections to the lateral hypothalamus (LH), stimulating Orexin A (OXA) release. OXA then interacts with OX1R at the PVN. This then facilitates an increased production and secretion of AVP to the periphery, where it causes increased blood pressure through various means including vasoconstriction, fluid reabsorption, and increased blood volume. CVO, circumventricular organs; LH, lateral hypothalamus; OXA, orexin-A; OX1R, orexin 1-receptor; PVN, paraventricular nucleus; and AVP, arginine vasopressin.

Hypertension is a major health concern that currently impacts a large majority of the general population. Despite its widespread prevalence, treatment options are not always sufficient to combat its detrimental impacts on an individual’s health. Furthermore, around half of those individuals diagnosed with hypertension are estimated to be classified as salt-sensitive (Weinberger et al., 1986; Weinberger, 1996). To this end, the present study sought to assess the role that orexin system hyperactivity may hold in the development of hypertension in the DOCA-salt hypertensive rat. We report four novel findings: (1) OX1R expression within the PVN of DOCA-salt rats is elevated compared to controls; (2) Chronic viral knockdown of OX1R function within the PVN of DOCA-salt rats reduces PVN AVP mRNA and protein expression; (3) Chronic viral knockdown of PVN OX1R function results in an attenuated plasma AVP concentration; and (4) The development of hypertension is significantly attenuated in the DOCA-salt rat following PVN OX1R knockdown. These results lead us to conclude that high salt intake causes activation of the CVOs. From here, neural projections are sent to cardiovascular relevant regions such as the PVN (Kawano and Masuko, 2010), in addition to the lateral hypothalamus (LH; Hahn and Swanson, 2010; Hurley and Johnson, 2014). These projections may directly and indirectly induce orexin A release to the PVN area. Upon binding with OX1R in the PVN, AVP production is stimulated, ultimately leading to the development of hypertension through numerous downstream physiological responses (vasoconstriction, fluid reabsorption, etc.; Figure 6). Furthermore, this effect can be significantly attenuated following OX1R knockdown in the PVN.

Arginine vasopressin plays a key role in physiological homeostatic regulation through interaction with its various receptor subtypes (V1, V2, and V3). Relevant to the current study, AVP interacts with V1 and V2 receptors to regulate smooth muscle vasoconstriction, and water reabsorption through reallocation of aquaporins in the collecting ducts of the kidneys (Thibonnier et al., 1998; Bankir et al., 2005; Treschan and Peters, 2006). Upon binding, downstream signaling cascades are activated, resulting in a net increase in blood pressure through vasoconstriction and volume retention. Importantly, AVP production within the PVN magnocellular neurons is initiated following a salt load due to interactions with circumventricular organs, implicating its actions in the pathogenesis of salt-sensitive hypertension. Furthermore, AVP has been observed to play a role in both Dahl-salt sensitive hypertension, in addition to DOCA-salt hypertension (Berecek et al., 1982a; Schenk and McNeill, 1992; Grillo et al., 1998; Huber et al., 2017). Interestingly, OX1R expressing neurons are co-localized with AVP producing neurons within the PVN (Backberg et al., 2002), indicative of some level of communication between the two mechanisms. Similarly, recent research from our lab (Huber et al., 2017) and others (Hernandez et al., 2018) has shown increased orexin system activity in both Dahl-salt sensitive and DOCA-salt rats.

The present study observed an increase in both central and peripheral AVP following DOCA-salt treatment, as would be expected. However, we also observed a concurrent increase in OX1R expression within the PVN, indicating a simultaneous increase in orexin activity and AVP production. To date, we are only aware of one other study that has assessed orexin system components’ expression in the DOCA-salt model (Hernandez et al., 2018). However, our previous work observed an increase in PVN AVP production following ICV injection of orexin-A (Huber et al., 2017), supporting the causative role of orexin signaling in AVP production and release to the periphery. As OXA has a higher binding affinity for OX1R, which is concentrated in the PVN, we posit that our results were primarily mediated through activation of the OX1R. This is indicative of a causative role of OXA binding in both the production, and systemic release of AVP which may point to a common pathway pertinent to SSH development. The increased orexin system components observed in the DOCA-salt rats, paired with increased AVP production following OXA ICV injection in normal SD rats (Huber et al., 2017) lends credence to our hypothesis that OXA-OX1R interactions facilitate increased AVP production and release. To further elucidate the modulatory role of orexin activity on AVP production and release, we injected an OX1R-shRNA viral vector, which effectively reduced OX1R expression within the PVN. In line with our hypothesis, this viral knockdown resulted in a concurrent decrease in PVN AVP expression, and release to the periphery. These findings outline the key role of OX1R in regulation of AVP production and release following a high-salt load in DOCA-salt rats. We elected not to test OX2R function in this model after previous work observed no impact of OX2R antagonism on AVP mRNA in primary neuronal cultures treated with OXA (Huber et al., 2017).

Orexin activity has been shown to play a key role in blood pressure regulation (Samson et al., 1999; Shirasaka et al., 1999; Schwimmer et al., 2010). Furthermore, antagonism of orexin through various means results in an attenuation of its pressor effects (Marques et al., 2011; Lee et al., 2013; Li et al., 2013; Huber et al., 2017). Indeed, microinjection of AVP elicits a similar increase in sympathetic outflow, while antagonism of the V1 receptor in the PVN attenuates SNA outflow and hypertension in high-salt intake rats (Ribeiro et al., 2015). In the DOCA-salt rat model, the hypertensive response is marked by an initial steep spike in blood pressure, followed by a more gradual increase and maintenance in the weeks following DOCA administration (Yemane et al., 2010). While there are some discrepancies in the etiology regarding the magnitude of impact in sympathetic outflow and DOCA-Salt hypertension development (de Champlain et al., 1968; Bouvier and de Champlain, 1986; Yemane et al., 2010), we elected to focus primarily on AVP as the key regulator, given its established role in the model. Our results observed the expected gradual increase in blood pressure for the 3-week period following DOCA-salt administration. However, chronic viral knockdown of PVN OX1R function attenuated the pressor response normally observed in the DOCA-salt rat model. Interestingly, it appeared as though the steep increase, as well as maintenance periods were equally dampened. While this may indicate some impact on sympathetic outflow, we cannot speak to this as we did not directly record nerve activity. However, Huber et al. (2017) previously observed that bilateral PVN microinjection of SB-408124, an OX1R antagonist, did not impact SNA outflow in Dahl-Salt sensitive rats, although it did drastically decrease mean arterial pressure in an acute setting. This relationship is indicative of the key role that orexin plays in salt-sensitive hypertension, specifically through modulation of AVP rather than sympathetic outflow. Our results confirm these at a chronic level, showing that viral knockdown of OX1R resulted in attenuation, but not complete amelioration, of hypertension development in DOCA-salt rats. While we cannot entirely rule out long-term modulation of sympathetic outflow, we presume that this attenuation occurred through the observed chronic attenuation of AVP production and release, and subsequent downstream activity. Further research utilizing sympathetic nerve recordings should be utilized to discern the impact orexin may have on sympathetic outflow in the DOCA-salt rat model.

It is worth noting the potential impact that other physiological systems may have had in preventing complete elimination of hypertension development. The RAS is a well-known hormonal blood pressure regulatory mechanism, and central RAS functioning, specifically in the PVN, has been shown to play a role in blood pressure regulation (Jensen et al., 1992; Bahner et al., 1995; Zhu et al., 2002; Chen et al., 2011). Angiotensin II (ANGII) receptors are densely localized in the PVN area (Pan, 2004), with some studies noting a higher consolidation of AT1 Receptors within parvocellular (pre-autonomic), rather than magnocellular PVN neurons (Lenkei et al., 1995; Oldfield et al., 2001). PVN microinjection of ANGII elicits a pressor effect presumably through both activation of sympathetic activity, as well as AVP release (Tsushima et al., 1994; Zhu et al., 2002; Khanmoradi and Nasimi, 2016). In the DOCA-salt rat model, increased ANGII receptor concentrations and binding have been observed within the PVN (Gutkind et al., 1988). Similarly, ANGII is elevated within the cerebrospinal fluid in the DOCA-salt rat model (Basting and Lazartigues, 2017). It is possible in our model that central ANGII activity may have had a role in the slight increase in blood pressure observed in our results. Our results show a significant dampening of blood pressure elevation following OX1R PVN antagonism, indicating that this pressor response may have been mitigated, at least in part, through the observed indirect reduction of AVP production through OX1R antagonism. Interestingly, despite a presumed intact effect of ANGII on parvocellular neurons and their subsequent innervation of pre-autonomic neurons, we did not observe a significant increase in blood pressure following PVN OX1R knockdown. This may be indicative of a lack of global sympathetic outflow in our specific model, which omits uninephrectomy (Kandlikar and Fink, 2011).

Similarly, neuroinflammation may have played a key role in the slight blood pressure increase in the OX1R knockdown rats. PICs have been observed to have an indirect impact on neural inflammatory responses through interactions with CVOs (Zhang et al., 2003; Wei et al., 2013, 2015), similar to the pathways utilized by ANGII. Central administration of proinflammatory cytokines, including Tumor Necrosis Factor alpha and Interleukin 1-Beta have caused a pressor response in normal SD rats (Shi et al., 2011; Wei et al., 2015). Research from our own lab has reported that proinflammatory cytokine expression is elevated in Dahl-Salt sensitive rats (Jiang et al., 2018). Further, we observed that OXA intracerebroventricular injection elicits substantial elevations in inflammation within the PVN (Fan et al., 2018), in addition to increased sympathetic outflow. However, we found that PVN injection of the OX1R antagonist, SB408124, significantly attenuated the sympathetic response to OXA (Fan et al., 2018). Our current findings may be in part due to a reduced neuroinflammatory response resulting from chronic OX1R downregulation. However, the DOCA-salt model is also marked by peripheral renal inflammation (Banek et al., 2019), which may have aided in the slightly elevated blood pressure despite central PVN OX1R knockdown. Unfortunately, we did not monitor central or peripheral inflammatory markers, and cannot elaborate on the role they may have played in the current study.

Additionally, we did not assess the influence of AVP production within the Supraoptic Nucleus (SON). The SON is a major area of AVP production (Treschan and Peters, 2006), and OX1R expressing neurons are co-localized with vasopressin producing neurons in this area (Backberg et al., 2002). The lack of OX1R knockdown within the SON may account for the partial blood pressure increase in the PVN OX1R knockdown rats. Further, this may also explain why we did not observe a significantly blunted plasma AVP release following PVN OX1R knockdown, as AVP from the SON may have been released to the periphery.

There are a few reasons why DOCA-salt treatment may activate the CVOs in our hypothesized model. First, increased plasma NaCl or increased osmolality may activate osmoreceptors located in the Organum Vasculosum of the Lamina Terminalis (OVLT) and the Subfornical Organ (SFO), which have projections to both the LH and PVN (Hahn and Swanson, 2010; Kawano and Masuko, 2010; Hurley and Johnson, 2014). Through this mechanism, signaling from the CVOs to the lateral hypothalamus may stimulate OXA release to the PVN, where it interacts with OX1R to elicit increased AVP production. Indeed, OVLT lesion partially attenuates the hypertensive response in DOCA-salt rats (Collister et al., 2018). Further, normalization of central and peripheral NaCl levels in DOCA-salt treated rats results in reduced blood pressure and sympathetic outflow (O’Donaughy and Brooks, 2006; O’Donaughy et al., 2006), while this was not observed in sham animals, signifying a key role for plasma NaCl in hypertension development. Second, circulating mineralocorticoids may directly impact activation of CVOs, since mineralocorticoid receptors are densely located in osmosensitive regions of the brain (Pietranera et al., 2004; Amin et al., 2005; Miller et al., 2013). Third, there is evidence that there may be synergistic activation of CVOs through both increased NaCl and circulating mineralocorticoids. Chronic DOCA treatment has been observed to augment the effects of increased NaCl, as decreasing circulating NaCl in DOCA-salt rats resulted in a greater blood pressure decrease when compared to rats treated with either DOCA or salt alone (O’Donaughy and Brooks, 2006). Evidence points toward cooperative activation of CVOs by both elevated NaCl and DOCA, which in turn send projections to both the lateral hypothalamus and PVN, potentially stimulating orexin system activity. However, we did not directly test the effect of CVO activation, and further efforts are necessary to validate our hypothesis.

The current study does yield some limitations. First, we elected not to perform a uninephrectomy in addition to the DOCA-salt treatment, as is often observed. However, we followed the previously established model exemplified by Kandlikar and Fink (2011), where uninephrectomy was not utilized in order to create a more gradual increase in blood pressure which more closely mimics that observed in human hypertension development. Furthermore, omitting the uninephrectomy allows us to better assess the direct impacts of orexin modulation by removing any of the cardiovascular implications that kidney removal incurs. Next, we recognize that the tail-cuff method is not the preferred standard of blood pressure measurement. Due to time and cost constraints, we were unable to perform radio telemetry measurements directly from arterial vessels. However, Feng et al. (2008) observed an adequate level of agreement between telemetry and tail-cuff blood pressure measurements, adding validity to our observed measurements. Additionally, we did take every precaution we could in order to reduce the stress on the animals while taking measurement (see Materials and Methods). Furthermore, we used the average of the weekly values for the first 2 weeks following DOCA-salt implantation, which reduces the error and deviations of daily measures. Further research should be conducted to repeat these findings using radio telemetry. We also recognize the importance of sex differences in blood pressure regulation. While we did not study females in this study, this also offers a future direction that requires exploration. Lastly, we note that use of a scrambled shRNA (AAV-Sc-shRNA) in addition to our AAV2-OX1RshRNA would strengthen our findings. However, in our previous unpublished data, in addition to a number of publications by Sun et al. (2008), scrambled AAV-Sc-shRNA injections were found to have no impact on blood pressure or other physiological parameters (Wang et al., 2012; Crosswhite et al., 2014; Chen and Sun, 2017). Because of these findings, we did not include an AAV-Sc-shRNA treatment group, although we do recognize that addition of a sham control would increase the rigor of the present study. Further studies are necessary to confirm our observed results.

In conclusion, our results indicate a key role for orexin in the development of SSH in the DOCA-salt rat model. We conclude that DOCA implantation paired with salt intake in SD rats results in heightened orexin system activation, primarily through OXA-OX1R binding. This heightened orexin activity then stimulates increased production of AVP within the PVN, and subsequent release to peripheral circulation, ultimately leading to the development of hypertension. This work furthers the role of orexin involvement in the pathogenesis of SSH and offers a potential mechanism that may be useful in eventual development of a pharmaceutical intervention to combat the negative impact of hypertension.

## Data Availability Statement

The raw data supporting the conclusions of this article will be made available by the authors, without undue reservation.

## Ethics Statement

The animal study was reviewed and approved by Michigan Technological University Institutional Animal Care and Use Committee (IUCAC).

## Author Contributions

JB and ZS conceived the research study and analyzed and interpreted the results. JB, HG, and ZS carried out all research protocols. JB, ZS, and Q-HC prepared and reviewed the final manuscript. All authors contributed to the article and approved the submitted version.

### Conflict of Interest

The authors declare that the research was conducted in the absence of any commercial or financial relationships that could be construed as a potential conflict of interest.
